# Genetic architecture of ideotype-related traits in middle American beans through single trait, multi-trait and epistatic genome-wide analyses

**DOI:** 10.1007/s00122-025-04924-w

**Published:** 2025-06-01

**Authors:** Henry A. Cordoba-Novoa, Valerio Hoyos-Villegas

**Affiliations:** 1 Department of Plant Science, McGill University, Montreal, Canada; 2 Department of Plant, Soil and Microbial Sciences, Michigan State University, 1066 Bogue St, East Lansing, MI USA

## Abstract

Common bean is one of the major legume crops for direct human consumption. The genetic improvement of common bean is a primary approach to increase crop adaptability to climate change conditions while maintaining productivity. A diverse panel of middle American beans was evaluated over three growing seasons (2021, 2022 and 2023) for agronomic traits considered in the crop ideotype such as flowering, maturity, pigment content, lodging, and yield. A study of the genetic regions controlling trait variation was carried out using single-trait and simultaneous (joint) multi-trait GWAS approaches. Additionally, genome-wide epistatic interactions were also analyzed. Several previously reported and novel regions were identified as significant for individual traits in the single and multi-year analyses with varying percentages of individual (7–52%) and collective (10 – 59%) phenotypic variance explained. In the single-trait and multi-year analyses, markers detected for lodging showed the highest average of percentage of variance explained (52%) followed by other traits with percentages between 11 and 19%. For yield, new loci were found with estimated effects between –96.19 to 90.96 kg/Ha in the multi-year data. In the multi-trait analyses, marginal loci on Pv02 and another on Pv04 were identified to have interaction effects on flowering and yield. A significant locus on Pv04 showed a common effect between lodging and maturity, characterized by several SNPs. Significant epistatic interactions were found along different chromosomes for all the evaluated traits, with some loci having interactions with multiple regions. In flowering, an interaction between loci on Pv01 and Pv04 explained up to 10.5% of the phenotypic variation, followed by interactions between Pv06 and Pv10 for chlorophyll b and between Pv03 and Pv08 for yield explaining around 6% of the trait variation. Multiple transcription factors were identified as candidate genes, particularly in the pairs of combinations of epistatic effects. Based on the homology analyses of the candidate genes, several showed potential roles in the genetic control of the agronomic traits, particularly for flowering, maturity, and yield. Our results demonstrate the applicability of various approaches for common bean and show a comprehensive and expanded panorama of the genetic basis of agronomic traits. The results provide new resources for crop improvement that can be leveraged in multiple approaches such as selection, modeling and predicting crop performance and genetic gain.

**Supplementary Information:**

The online version contains supplementary material available at 10.1007/s00122-025-04924-w.

## Introduction

Common bean (*Phaseolus vulgaris* L.) is the most important cultivated legume for human consumption and a staple crop in both developed and developing countries (OECD 2019). To maximize productivity in tropical and subtropical regions where beans are cultivated, varieties with desirable agronomic traits are needed. A crop ideotype defines an idealized model that combines morphological and physiological traits to maximize yield and quality under a certain environment (Donald 1968). First adopted by Adams (1982), and later expanded by Acquaah et al. (1991), the common bean ideotype describes the characteristics a plant must have to increase productivity. Some of the main traits considered in the ideal plant are short flowering and maturity periods that maximize resource use and harvest tasks, and an upright architecture that improves light interception and minimizes losses due to soil-borne diseases and mechanical harvest. The improvement of these individual traits, which are less genetically complex compared to yield, will contribute to the overall performance of the plan with a potential impact on crop yield.

Among other factors, the successful improvement of traits in breeding programs heavily depends on effectively selecting parents and progeny that accumulate beneficial alleles to achieve the ideal phenotype. Over the last decades, genome-wide association studies (GWAS) in plants have shed light on the genetic control of multiple yield-related traits. These results have contributed to improving the selection of potential lines through Marker-Assisted Selection (MAS) or, more recently, through genomic prediction (GP) and modeling by including information on relevant loci in the analyses (O’Connor et al. 2020; Kaler et al. 2022; Lin et al. 2023; Zhang et al. 2023a).

In common bean, several studies have identified QTLs involved in the control of agronomic traits in Andean and Middle American bean populations (Kamfwa et al. 2015; Moghaddam et al. 2016; Hoyos‐Villegas et al. 2017; Diaz et al. 2020). Depending on the location, new validations lead to the discovery of additional loci or genes that may contribute to the observed variation in the traits (Mohammadi et al. 2020; Sallam et al. 2023; Sahito et al. 2024). Despite the contributions and benefits of GWAS, some limitations need to be overcome. GWAS can be affected by confounding factors such as population structure and relatedness, which need to be considered in the models. Single-locus models such as mixed linear models (MLM), and multi-locus models, such as fixed and random model circulating probability unification (FarmCPU) and Bayesian information and linkage disequilibrium iteratively nested keyway (BLINK) have been developed with varying levels of success in analyzing diverse complex traits, where multi-locus has shown to be superior (Merrick et al. 2022; Sahito et al. 2024). The differences in effectiveness of single- and multi-locus GWAS models may be attributed to trait architecture, population structure, and marker density. For instance, when evaluating leaf anatomical traits, Narawatthana et al. (2023) found that multi-locus models may have produced a higher number of false positives compared to single-locus models. This highlights the importance of testing several GWAS models to find the method that best fits the genetic architecture of the trait and the studied population.

Other challenges in GWAS include that GWAS only explains a fraction of the heritability (Brachi et al. 2011), ultra-rare variants are not easily detected, not all determinants of traits can be identified, the causal variant is usually not distinguished, and genotype interactions are not routinely analyzed (Tam et al. 2019; Tibbs Cortes et al. 2021). Some attempts to improve and expand the scope of GWAS include the use of diverse populations, the simultaneous analysis of related traits, the adoption of new gene model actions, and the study of genotype-by-genotype (epistasis) and genotype-by-environment (G × E) interactions.

In the analysis of multiple traits, co-localization of significant markers independently identified in single-trait GWAS has been one of the main tools used in different crops, including beans (Kamfwa et al. 2015; Jaiswal et al. 2016; Saballos and Williams 2024). Korte et al. (2012) developed a joint multi-trait mixed model (MTMM) that simultaneously considers the variance observed within and between correlated traits. MTMM analysis has allowed the identification of regions and genes with effects on multiple traits (Oladzad et al. 2019a; Malik et al. 2022).

For epistatic interactions, Li et al. (2022) recently proposed a three-variance multi-locus random-SNP-effect mixed linear model (3mrMLM) method to model QTN-by-QTN interactions (QQIs). The method is based on the estimation of the genotypic effects for pairs of QTNs depending on the allelic state (homozygous alternative, recessive, and heterozygous (AA, Aa, aa)) and the partition of the effects into additive or dominance effects using a one-way analysis of variance. The estimated combination effects will consider when one or both QTNs have an additive- or dominance-modeled effect, as well as the combination (Li et al. 2022). In an independent study, Zhang et al. (2023a) used the 3mrMLM method to identify epistasis in traits related to the saline–alkali tolerance in rice. The authors found significant QQIs and associated candidate genes that showed differential expression under control and salt stress conditions. Furthermore, the inclusion of QQIs in genomic prediction models showed higher prediction abilities, demonstrating the ability of the method for gene mining and selection efforts in breeding programs.

Here, we conducted a study on the genetic basis of agronomic traits in common bean to contribute to current and future breeding efforts for the crop. The main goal was to leverage multiple approaches for GWAS that allowed us to expand our knowledge on the genetic control of ideotype-related traits. For GWAS, we implemented single-trait, joint multi-trait, and epistatic models to identify genomic loci involved in the control of single traits, loci with pleiotropic effects in more than one trait, and regions interacting across the genome (epistasis), respectively. With the identified loci, we analyzed potential candidate genes and alleles for introgression and future selection in breeding programs.

## Materials and methods

### Common bean diversity panel and field experimental conditions

The common bean Mesoamerican Diversity Panel (MDP) from BeanCap (Moghaddam et al. 2016) was evaluated during three growing seasons between 2021 and 2023 at the Emile A. Lods Agronomy Research Centre at McGill University, Montreal, Quebec, Canada following local agronomic standard practices for pest and disease management. The MDP consists of modern dry bean cultivars from races Durango–Jalisco and Mesoamerica with different US market classes represented (Moghaddam et al. 2016). A total of 285 genotypes were grown in plots of four, five-meter-long rows with 75 seeds per row and a row spacing of 0.76 m. The experiments were laid out in a randomized complete block design with 3 replications. Plots were maintained with standard agronomic practices for fertilization and pest, disease, and weed control.

### Phenotypic evaluation of agronomic traits

The MDP was evaluated during the growing season and data was collected only from the two middle rows to avoid border effects. Days to flowering (DTF), days to maturity (DTM), lodging and leaf pigment concentration (chlorophylls and carotenoids) were measured in two seasons and yield during three growing seasons.

DTF were visually recorded per plot in 2021 and 2023 as the number of days from planting when 50% of the plants had 50% open flowers. DTM were measured when 50% of the plot reached physiological development and senescence (R9). Lodging was scored at maturity on a 1 to 10 scale where 1 indicates 100% plants standing erect and 10 indicates 100% plants flat on the ground. DTM and lodging were recorded in both years 2021 and 2022.

Leaf pigments were measured before flowering (stage V10) and at flowering (R1) in 2021 and only at flowering in 2022 according to Singh et al., (2013). Briefly, five fully expanded and mature leaves from the middle part of the plant canopy were randomly collected in the field and five 0.20 cm^2^ leaf disks were placed in black microcentrifuge tubes containing 1300 µL of 95% ethanol using a MIDCO 2390 Tissue J punch. Tubes were incubated at room temperature for 24 h, vigorously shaken with vortex for 30 s, and 200 µL of supernatant were transferred to Corning® 96-well cell culture plates. Absorbance was measured at 664, 648, and 470 nm with a TECAN Infinite M200 microplate reader. Total chlorophyll (totChl), chlorophyll A (ChlA), chlorophyll B (ChlB), and carotenoids concentrations were estimated using the equations of Lichtenthaler, (1987) and expressed in micrograms per milliliter (µg/mL). For further analyses, only measurements taken at flowering were included to keep the datasets balanced and comparable between years.

The two data rows of each plot were mechanically harvested with a Wintersteiger Classic Plus plot combine. For each plot, weight was recorded, and moisture was measured with a DICKEY-john® GAC™ 2500-INTL Grain Moisture Analyzer. Yield per plot calculated for the three years (2021 – 2023) in Kg per Ha considering plot moisture, and a standard moisture of 18% according to the following formula.

$${\text{Yield}} \left( {Kg/Ha} \right) = \left( {\frac{{{\text{Plot}} {\text{weight}} \left( {Kg} \right)}}{{{\text{plot area}} \left( {Ha} \right)}}} \right)*\left( {\frac{{100 - {\text{plot}}\;{\text{moisture}}}}{{100 - {\text{standard}}\;{\text{moisture}}}}} \right)$$

### Data analysis

Experiments were planted in a randomized complete block design (RCBD) with three replicates for a total of 855 plots. Data were verified for normality using Shapiro–Wilk test, missing and extreme values. Best Linear Unbiased Predictor (BLUP)-corrected means were estimated for each genotype in each year by fitting a mixed-effects model using the REML method in the *lme4* package in R (Bates et al. 2015). For the single-year analysis, the model was as follows:

$$Y_{ijk} = \mu + \tau_{i} + \beta_{j} + e_{ijk}$$

where ${\tau }_{i}$ is the random effect of the *i-th* genotype, ${\beta }_{j}$ is the random effect of the *j-th* block, and ${e}_{ijk}$ is the experimental error associated with observation *ijk.*

For the combined years analysis, years were considered as environments in the following model:

$$Y_{ijkm} = \mu + \tau_{i} + \upsilon_{k} + \left( {\tau \times \upsilon } \right)_{ij} + \beta_{j\left( k \right)} + e_{ijkm}$$

where ${\tau }_{i}$ is the random effect of the *i-th* genotype, ${\upsilon }_{k}$ is the fixed effect of the *k-th* environment (year), ${(\tau \times \upsilon )}_{ij}$ is the interaction effect between the *i-th* genotype and *k-th* environment, ${\beta }_{j(k)}$ is the random effect of the *j-th* block nested within environment *k*, and ${e}_{ijkm}$ is the experimental error associated with observation *ijkm*.

For each trait, broad-sense heritability (H^2^) was estimated in the multi-year analysis by:

$$H^{2} = \frac{{\hat{\sigma }_{g}^{2} }}{{\hat{\sigma }_{g}^{2} + \frac{{\hat{\sigma }_{{\left( {\tau \times \upsilon } \right)}}^{2} }}{k} + \frac{{\hat{\sigma }_{e}^{2} }}{k*n}}}$$

where ${\widehat{\sigma }}_{g}^{2}$ is the variance attributed to the genotype, ${\widehat{\sigma }}_{(\tau \times \upsilon )}^{2}$ the genotype × environment interaction variance, ${\widehat{\sigma }}_{e}^{2}$ the error variance,* k* and n are the number of environments and replicates in each environment, respectively.

Phenotypic correlations between traits were calculated using the BLUP-corrected means and the Pearson’s correlation method in the *cor* function in base R v4.2 (R Core Team 2022). Genetic correlations were estimated using META-R (Alvarado et al. 2020) as follows:

$$\rho_{g} = \frac{{\overline{{\sigma_{{g\left( {jj^{\prime}} \right)}} }} }}{{\overline{{\sigma_{g\left( j \right)} \sigma_{{g\left( {j^{\prime}} \right)}} }} }}$$

where ${\rho }_{g}$ is the genetic correlation between two traits, $\overline{{\sigma }_{g\left(j{j}{\prime}\right)}}$ is the aritmetic mean of the genotypic covariances and $\overline{{\sigma }_{g\left(j\right)}{\sigma }_{g\left({j}{\prime}\right)}}$ is the mean of the pairwise geometric means the genotypic variances of the traits (Ortiz et al. 2021).

### Single and multi-trait genome-wide association analysis

The common bean MDP was previously genotyped using low-pass sequencing, Genotype-by-Sequencing (GBS), and the Illumina BeadChips BARC-BEAN6K_1 and BARCBEAN6K_2 (Moghaddam et al. 2016). SNP Data are publicly available on the GitHub folder (see data availability statement). SNPs with a MAF < 0.05 were filtered out. However, SNPs with 0.02 < MAF < 0.05 were retained to explore *marginal* significant associations in an independent analysis. This lower MAF threshold was used to facilitate the discovery and exploration of potential low-frequency alleles (Ray et al. 2015). However, it should be analyzed and interpreted with caution and alongside candidate genes information. Genotypic data were used to assess population structure using Principal Component Analysis (PCA) in Tassel (Bradbury et al. 2007).

Single-trait Genome-Wide Association (st-GWA) analyses were performed in GAPIT v3 (Wang and Zhang 2021) using single and multi-locus models and the BLUPs estimated as described above. False positives due to population structure were controlled by using principal component analysis (PCA) and kinship as parameters in the GWAS models. Kinship was calculated with the VanRaden method (VanRaden 2008) integrated into GAPIT.

Mixed Linear Model (MLM), Settlement of MLM Under Progressively Exclusive Relationship (SUPER), Fixed and random model Circulating Probability Unification (FarmCPU), and Bayesian information and LD Iteratively Nested Keyway (BLINK) were compared based on the model adjustment in the QQ-plots. FarmCPU was used for the reported results. FarmCPU includes kinship (K) and population structure (Q) as covariates in the model, but also controls the confounding between the testing marker and both K and Q by allowing the fixed and random effects models perform separately, while adjusting K (Liu et al. 2016). To find genomic loci with pleiotropic common or interaction effects between pairs of traits, multi-trait GWAs (mt-GWA) were performed following the multi-trait mixed model (MTMM) approach and scripts developed by Korte et al. (2012) using BLUP-corrected means. The MTMM model is based on the theory behind the mixed models described by Henderson (1984) where, when analyzing two traits, the phenotypic covariance matrix is defined as:

$${\text{cov}} \left( {y_{1} ,y_{2} } \right) = \sigma_{g1} \sigma_{g2} \rho_{g} K\; + \;\sigma_{e1} \sigma_{e2} \rho_{e} I$$

where ${y}_{1}$ and ${y}_{2}$ are the values for traits 1 and 2, ${\sigma }_{g}$ and ${\sigma }_{e}$ are the genotypic and error variance associated with traits 1 or 2, respectively, ${\rho }_{g}$ captures the genetic correlation between the two phenotypes and ${\rho }_{e}$ includes the correlation between traits due to shared environments or error sources. K is a *n* × *n* kinship matrix and *I* is the identity matrix identity matrix. For more information on the method’s implementation, see Korte et al. (2012).

The percentage of the variance explained by the individual and collective significant SNPs in each year was calculated using the coefficient of determination (R^2^) of a linear regression model fitted to the data. The model included three first principal components (PC) from the PCA and the estimated effect of each marker according to one of three gene action models: additive, dominance of the reference allele, and dominance of the alternative allele. The marker effect was estimated using the GWASpoly package implemented in R with a diploid setting (Rosyara et al. 2016).

For both st-GWA and mt-GWA, the threshold for declaring a significant association was corrected for multiple testing using the False Discovery Rate (FDR) criterion with the Benjamini and Hochberg (1995) method.

### Genome-wide epistasis analysis

A three-variance-component multi-locus random-SNP-effect mixed linear model (3VmrMLM) approach (Li et al. 2022) was used to identify quantitative trait nucleotides (QTNs) with epistatic effects (QTN-by-QTN interactions—QQIs). As before, BLUP-corrected means from the combined multi-year experiment were used. Testing all the possible pairwise interactions between the ~ 200K available SNPs would be computationally demanding. Therefore, the genotypic dataset was filtered down by removing SNPs with MAF < 0.05 and by thinning SNPs with a 10,000 nucleotides distance using Tassel v5 (Bradbury et al. 2007). SNPs were further pruned based on linkage disequilibrium (LD) with the ‘indep-pairwise’ option in Plink, using a window size of 50Kb, a step size of 50 and a squared correlation (r^2^) threshold of 0.3, as previously reported for common bean (Keller et al. 2020). Our purpose was to obtain a highly reduced, but evenly distributed and representative dataset for the epistatic analysis. Therefore, the r^2^ threshold was picked to increase the dataset reduction and favor the computational analysis. After pruning, a set of 1,978 SNPs were used for the analyses run in R and the ‘IIIVmrMLM’ package using the function ‘epistasis’, with the following settings: SearchRadius = c(0,1); svpal = c(0.05,0.05); and sblgwas_*t* = − 1 (default). With this dataset, a total of 1,955,253 genome-wide pairwise comparisons were analyzed. The method provides suggested interactions if the LOD score is > 3 and significant interactions when the *p* value of the interaction is below the Bonferroni-corrected cutoff (*p* < 0.05). Unlike the FDR correction applied in the st- and mt-GWAS, for the epistasis analysis a more stringent multiple-testing correction is recommended to identify QQIs with enough confidence. Additionally, the method already suggests a more relaxed threshold (LOD > 3) for potential interactions that must be analyzed carefully and within a biological context.

### Candidate gene analysis

Candidate genes were analyzed in a 100 Kbp SNP-centered window using the genomic annotation files from the *Phaseolus vulgaris* reference genome v2.1 (Schmutz et al. 2014) in Phytozome 13 (https://phytozome-next.jgi.doe.gov/info/Pvulgaris_v2_1). The window was defined in accordance with previous GWAS using the MDP (Moghaddam et al. 2016) and based on the genome-wise LD decay pattern calculated using a non-linear regression of the expectation of the parameter r^2^ and the distance between each pair of markers (Hill and Weir 1988; Remington et al. 2001). A custom script was used to retrieve the candidate genes within the window, and genes were further selected based on their annotation, level of expression in different common bean tissues (O’Rourke et al. 2014), and the homology with *Arabidopsis thaliana* gene models (TAIR10; Berardini et al. 2015).

## Results

### Agronomic traits analysis

**Fig. 1:**
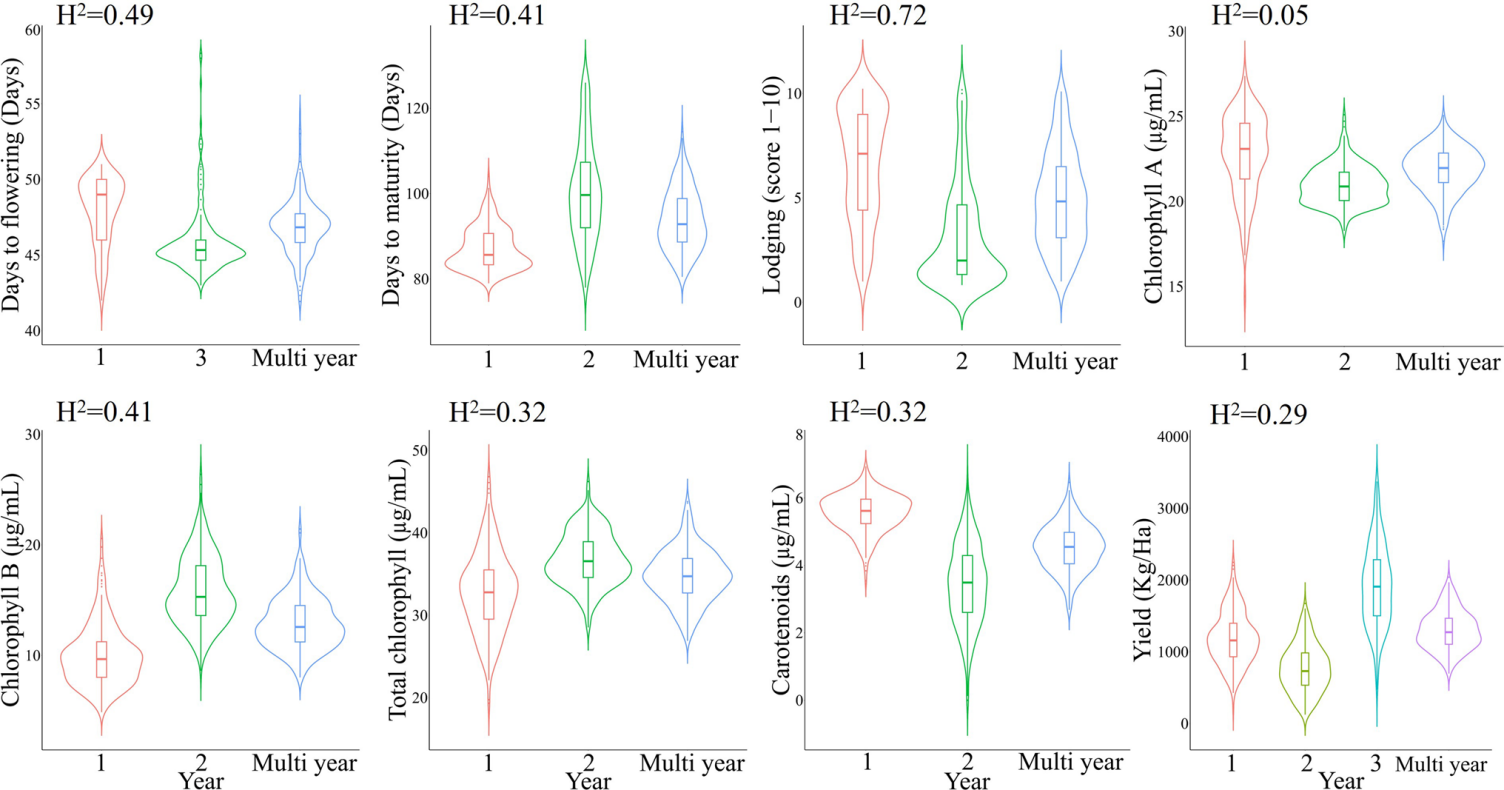
Phenotypic distribution of the BLUP-corrected means of the evaluated agronomic traits during different years and multi-year analysis. Broad-sense heritability (H^2^) for each trait is included in the top part of each panel

Days to flowering, days to maturity, lodging and pigments had similar variations and means in the first and second years of evaluation. However, yield showed more variation among years, with higher yields in the third year and lower minimum yields in the second year (Fig. 1). In the ANOVA, the blocking factor (replicate) was significant (*p* < 0.05) for DTF, DTM, pigments and yield in 2021; for lodging in 2022; and for lodging, pigments, and yield in the combined analysis (data not shown).

Strong significant correlations based on BLUP-corrected means were observed between leaf pigments. ChlA and ChlB were positively correlated with the totChl concentration (*r* = 0.76 and 0.93, respectively), but totChl and ChlB were negatively correlated with carotenoids concentrations (*r* = − 0.37 and − 0.64, respectively). Genetic correlations followed the same trend as the correlations based on BLUP corrections, but with higher values (Figure S1). Although not strong (*r* = − 0.3 to − 0.13)*,* yield was negatively correlated with lodging, ChlA and B, and totChl, while positively correlated with DTF (*r* = 0.17; Figure S1).

The calculation of broad-sense heritability (*H*^*2*^, Fig. 1), resulted in the highest heritability for lodging (0.72), followed by DTF (0.49), and DTM and ChlB (0.41). TotChl and carotenoids had similar *H*^*2*^ (0.32). ChlA had a considerably lower *H*^*2*^ value (0.05).

### Single-trait genome-wide association studies

Agronomic traits were independently analyzed in each year and in a combined analysis across years using the corrected means from a multi-environment model where an environment denotes a year. In the single-trait GWAS using FarmCPU, the observed *p* values in the QQ plot were distributed along the expected diagonal with only noticeable deviations in the right tail (highest –log(*p*)) across different datasets (Figure S3A-G).

For days to flowering (DTF), two significant and one marginal SNPs were detected on Pv01, Pv04 and Pv09 in the first year (Table 1, Table S1-2) explaining between 6and 8% of the phenotypic variance (PV) individually (R^2^) and up to 10% for only the significant SNPs together. Five significant peaks were identified in the third year with major signals on Pv11, Pv02, and Pv01 with a 14% of PVE (Table 1; Table S1). In the combined analysis, one significant and three marginal loci were detected on Pv01, Pv02, Pv08 and Pv11, where the SNP S11_50734871 was only 4 bp away from the SNP S11_50734875 identified in the analysis of the third year (Table 1; Table S1-2). However, the SNP found in the third year had an estimated effect of + 1.39 in contrast to -0.93 for the SNP from the common analysis, but both explained around 11% of the phenotypic variance.

**Table 1:** Most significant marker-trait associations (Highest –Log_10_(*p*)) identified in single-trait GWAS for individual years and the multi-year data

Trait	Year	Marker ID	Pv Chr	Position (bp)	-log_10_(*p*)	MAF	Estimated Effect	*R*^2^	Joint *R*^2^ *
Flowering (Days)	1	S09_8297313	9	8,297,313	6.72	0.05	− 0.23	0.07	0.10
		S01_41094580	1	41,094,580	6.33	0.05	0.23	0.08	
	3	S11_50734875	11	50,734,875	10.66	0.05	1.39	0.11	0.14
	Multi-year	S11_50734871	11	50,734,871	6.07	0.05	− 0.93	0.11	-
Maturity (Days)	1	S04_39063963	4	39,063,963	8.18	0.21	− 1.56	0.12	0.20
		S02_25857841	2	25,857,841	7.45	0.34	1.12	0.09	
	2	S01_1998312	1	1,998,312	12.59	0.25	− 5.11	0.12	0.28
		S03_32405157	3	32,405,157	12.31	0.43	4.11	0.14	
	Multi-year	S01_34529676	1	34,529,676	8.44	0.14	2.90	0.16	0.29
		S01_35451714	1	35,451,714	7.32	0.40	− 2.35	0.11	
		S03_29601114	3	29,601,114	7.31	0.44	− 3.16	0.11	
		S04_14884840	4	14,884,840	7.08	0.38	− 2.05	0.13	
		S04_40907424	4	40,907,424	6.97	0.13	− 2.35	0.13	
Lodging (score 1–10)	1	S09_35943555	9	35,943,555	16.35	0.49	0.89	0.48	0.54
		S06_18744301	6	18,744,301	6.89	0.40	− 0.63	0.47	
	2	S08_12832727	8	12,832,727	11.58	0.15	− 0.12	0.47	0.57
		S04_4185943	4	4,185,943	6.79	0.31	− 0.08	0.51	
	Multi-year	S01_38180559	1	38,180,559	11.78	0.05	− 0.10	0.46	0.59
		S04_4185944	4	4,185,944	8.10	0.31	0.09	0.51	
		S06_18742429	6	18,742,429	7.61	0.40	− 0.06	0.47	
Total Chlorophyll (µg/mL)	1	S06_23899103	6	23,899,103	8.02	0.42	1.17	0.16	0.44
		S03_42619117	3	42,619,117	6.94	0.49	1.46	0.13	
	2	S11_43201973	11	43,201,973	10.49	0.35	1.22	0.10	0.22
		S04_25532988	4	25,532,988	6.57	0.30	− 1.21	0.12	
	Multi-year	S07_1457666	7	1,457,666	9.09	0.37	0.70	0.11	0.29
		S08_62060162	8	62,060,162	9.05	0.15	− 1.01	0.14	
		S08_8912268	8	8,912,268	7.69	0.18	− 1.02	0.11	
		S10_34272441	10	34,272,441	6.73	0.38	1.25	0.11	
		S11_50893830	11	50,893,830	6.11	0.44	− 0.99	0.16	
Chlorophyll B (µg/mL)	1	S08_20435750	8	20,435,750	7.90	0.44	1.37	0.14	0.22
		S01_16088392	1	16,088,392	6.52	0.50	1.62	0.16	
	2	S08_41560414	8	41,560,414	9.52	0.25	0.06	0.14	0.23
		S06_1824892	6	1,824,892	8.95	0.23	− 0.09	0.16	
	Multi-year	S02_1604363	2	1,604,363	11.05	0.46	− 0.60	0.16	0.39
		S04_21552993	4	21,552,993	10.56	0.26	1.62	0.18	
		S04_31012356	4	31,012,356	8.70	0.32	0.52	0.16	
		S05_40619283	5	40,619,283	8.41	0.10	0.68	0.15	
Carotenoids (µg/mL)	1	S07_3329188	7	3,329,188	10.42	0.27	0.02	0.16	0.16
		S01_12584337	1	12,584,337	9.40	0.29	0.03	0.15	
	2	S01_8441723	1	8,441,723	8.48	0.27	0.45	0.17	0.17
		S08_11024883	8	11,024,883	7.71	0.47	0.57	0.16	
	Multi-year	S05_28829663	5	28,829,663	8.49	0.41	0.21	0.19	0.32
		S05_40685667	5	40,685,667	7.00	0.39	− 0.16	0.18	
		S07_29306628	7	29,306,628	6.81	0.41	0.27	0.20	
		S07_31201555	7	31,201,555	6.71	0.37	− 0.20	0.19	
		S11_35698632	11	35,698,632	6.19	0.13	− 0.19	0.16	
		S11_49484186	11	49,484,186	6.16	0.05	0.29	0.19	
Yield (Kg/Ha)	1	S09_33477028	9	33,477,028	8.20	0.39	103.70	0.14	0.21
		S01_17315579	1	17,315,579	8.06	0.09	168.43	0.137	
		S01_28269387	1	28,269,387	7.39	0.17	127.73	0.129	
	3	S04_47063565	4	47,063,565	13.32	0.43	− 254.94	0.13	0.14
		S05_31748737	5	31,748,737	8.78	0.30	216.11	0.13	
	Multi-year (3 years)	S02_37292928	2	37,292,928	10.57	0.38	80.24	0.17	0.32
		S03_15064177	3	15,064,177	9.50	0.39	− 66.07	0.16	
		S04_2265016	4	2,265,016	7.87	0.29	− 45.33	0.13	
		S06_1757726	6	1,757,726	7.03	0.42	80.03	0.19	
		S08_4969454	8	4,969,454	6.10	0.39	− 96.19	0.17	
		S10_11495606	10	11,495,606	5.78	0.47	68.23	0.15	
		S11_16896781	11	16,896,781	5.76	0.36	90.96	0.13	

For days to maturity (DTM), a total of 18 significant SNPs were identified in the single and multi-year analysis (Table S1). In the combined analysis, the SNP S01_34529676 was common with year two and had a maximum estimated effect of 5.4 days, explaining 16% of the phenotypic variance (Table 1). Moreover, SNPs S04_39063963 (R2 = 0.12) from the first year and S04_40907424 (R2 = 0.13) from the combined analysis were only 1.8 Mbp apart with effects of − 1.6 and − 2.3 days, respectively (Table 1 and Table S1).

For Lodging, 17 SNPs were detected as significant and tow as marginal for the three analyses with similar percentages of individual PVE (R^2^ around 0.48 for each SNP) and with a mean of 57% PVE when considered together within each year (Table 1; Table S1-2). In year 1, S09_35943555 was highly significant with an effect of 0.9 units and in year 2, S08_12832727 had the highest -Log(*p*) value (11.6) with an effect of − 0.12. For the multi-year analysis, S04_4185944 and S06_18742429 co-localized with S04_4185943 and S06_18744301 from year 2 and year 1, respectively (Table 1).

In leaf pigments, no significant SNPs were detected for ChlA. For ChlB, 19 significant and one marginal SNPs were identified, with most of them in the combined analysis (Table 1; Table S1-2). The stronger signals were obtained for S08_20435750, S08_41560414, and S04_21552993 in years 1, 2 and multi-year, respectively, each SNP explaining around 14% of the phenotypic variance. In Years 1 and 2, the significant SNPs collectively represented 23% of the variation, whereas in the multi-year analysis, they accounted for up to 39% (Table 1 and Table S1). For totChl, there were a total of 26 significant SNPs, where SNPs on Pv06 and Pv11 had the highest -Log(*p*) values. The SNP S11_50893830 was common between the year 1 analysis and the multi-year with the highest percentage of explained variance (16%). The SNPs S04_25532988, S06_1824892, S08_20435750, and S08_41560414 were commonly identified for ChlB and totChl concentrations in different years (Table S1). Together, the significant SNPs identified in year 1 explained a higher PV (44%) compared to year 2 (22%) and multi-year (29%). For carotenoids, 11 significant and one marginal SNPs were detected mainly on Pv01, Pv05, Pv07 and Pv11. In the multi-year analysis, S07_29306628 and S11_49484186 (MAF = 0.047) had positive effects of around 0.27 units, and S07_31201555 and S11_35698632 had negative effects of − 0.21 units, each SNP explained around 17% of the phenotypic variation with a joint PVE around 17% for year 1 and 2, and 32% for the multi-year dataset (Table 1). However, no co-localized SNPs across environments (years) were identified for carotenoids concentration.

For yield, eight significant and two marginal SNPs were identified in the first year, five significant in the third year and seven in the multi-year analysis (Table S1). No significant or marginal SNPs were identified for the second season. Across all years, S04_47063565 in year 3 had the highest -Log(*p*) (13.3) explaining 13% of the variance and an effect of − 254.94 units, followed by S02_37292928 (10.6) from the three-year analysis which explained 17% and had an effect of 80.24 units (Table 1). Finally, S06_14648484 (8.37) in the first year was the most significant (*R*^2^ = 0.16) with an effect of—372.29 units. Interestingly, four SNPs detected in the three-year analysis (S02_37292928, S03_15064177, S06_1757726, and S08_4969454) were also identified when analyzing the different years by pairs in different combinations (Table S1). In the combined analysis, S08_4969454 and S11_16896781 had the highest absolute effect on yield around 90 kg/Ha. When simultaneously considered, the significant SNPs explained from 14% (year 1) to 32% of the yield variation (multi-year).Genome-wise, the LD decay for the evaluated population was 136.3 Kb. Candidate genes were identified for each individual trait (Table S4 – S8) and potential roles in the control of agronomic traits were analyzed (see discussion for GWAS candidate gene analyses). In the PCA for population structure, two subpopulations were observed (Figure S2).

### Multi-trait genome-wide association studies

**Fig. 2:**
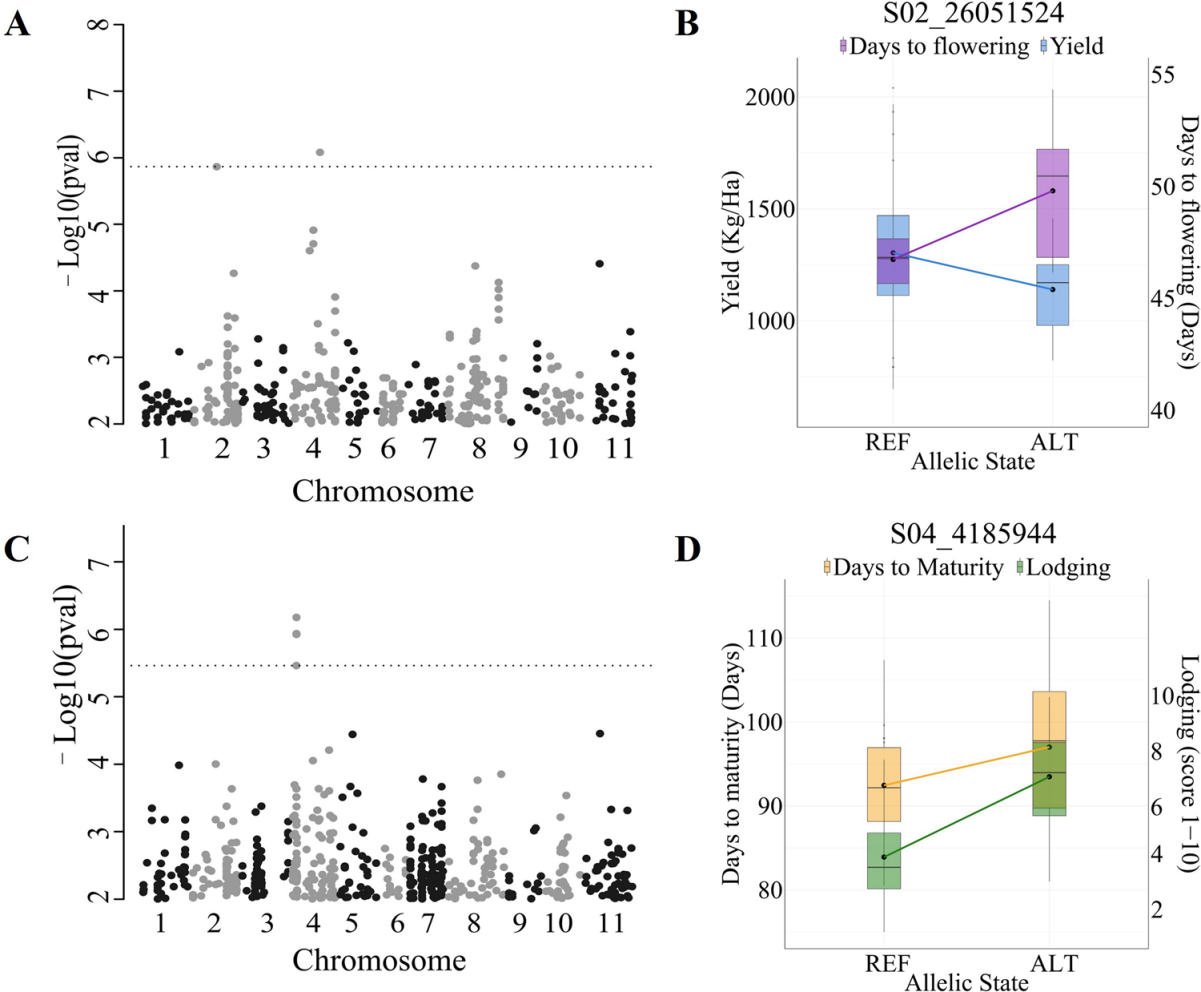
Multi-trait GWAS for yield and DTF (**A**), and example of interaction effect observed for yield and DTF for the SNP with the highest effect (**B**). Analysis for DTM and lodging (**C**) and common effect between DTM and lodging for the most significant SNP (**D**). Significant associations are above the FDR corrected threshold (*q* < *0.05*). REF denotes the reference allele and ALT is the alternative allele in the variant calling against the common bean reference genome v2.1. In B and D, black dots denote the mean and horizontal bars the median of the BLUP-corrected phenotypes for each allelic state combination

BLUP-corrected means from multi-year data were used to identify genomic loci with pleiotropic effects between pairs of traits. For DTF, the S04_29597528 [C/T] and S02_26051524 [C/T] loci had a *marginal* (MAF = 0.04) interaction effect with yield, each one explaining 12% of the yield variance based on the R^2^ (Fig. 2A, Table 2, S3). Interestingly, the SNP on Pv02 was 154 Kbp from the SNP S02_25897197 [A/G] detected as significant for yield in the first year. The change from the reference to alternative alleles in S02_26051524 causes a reduction in yield of 164.2 kg/Ha (from 1303.8 kg/Ha to 1139.6 kg), but increases the DTF from 47 to 50 days (Fig. 2B). A similar behavior between DTF and yield was observed for the SNP on Pv04 (Figure S4).

**Table 2:** Marker-trait associations found in a Multi-trait GWA using combined multi-year data BLUPs. Trait 1 and 2 refer to the traits included in the first column (traits) as they appear

Traits	Relationship between traits	Marker ID	Pv Chr	Position (bp)	-Log10(p)	MAF	Estimated effect Trait 1	Estimated effect Trait 2	R^2^ Trait 1	R^2^ Trait 2
Days to maturity (Days) and Lodging (score 1–10)	Common effect	S04_4185944	4	4,185,944	6.18	0.31	4.57	3.11	0.13	0.5
		S04_4180303		4,180,303	5.93	0.32				
		S04_4180312		4,180,312	5.92	0.32				
		S04_4180313		4,180,313	5.92	0.32				
		S04_4143864		4,143,864	5.46	0.32				

Regarding common effects, the same peak on Pv04 (S04_4185944) identified for single-trait GWAS for lodging, also showed a significant common effect with DTM (Fig. 2C, Tables 1 and 2). When the reference allele is present, the average DTM and lodging are 92.4 and 3.9, respectively, but when both alleles are alternative [TT], DTM increases to 97 and lodging to 7.0 (Fig. 2D). Based on the model adjustment, S04_4185944 explained 50% of the variation in lodging and 13% of the variation in maturity. Candidate genes for the interaction and common effects between pairs of traits were also identified for each significant SNP (Fig. 2, S4; Table S9).

### Genome-wide epistasis analysis

**Fig. 3:**
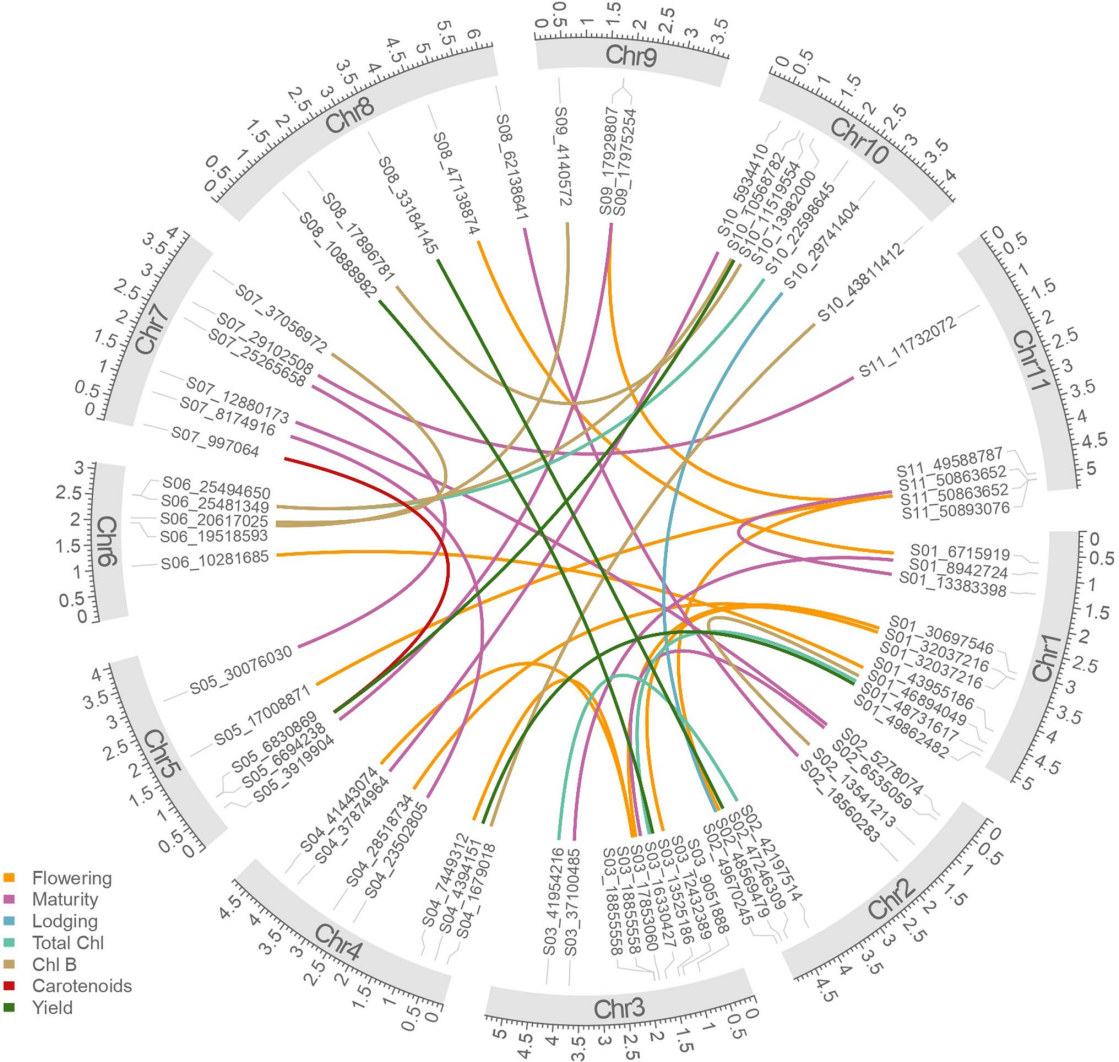
Significant QTN-by-QTN interactions (QQIs) detected for the agronomic traits. The lines connecting different loci represent interactions for different agronomic traits coded as colors

Multiple genomic loci with significant epistatic effects were detected for the evaluated agronomic traits (Fig. 3, Figure S5, and Table S10). Table 3 summarizes the results for the mont significant interactions, including the PVE, valuable allelic combination, and the difference of the observed mean phenotype for that allelic combination and the overall mean of the trait (BLUP-corrected). The proposed “valuable” allelic combination is the allelic states for each interaction that reduces or increases the phenotype depending on the trait (Table 3, and Table S11). For flowering, 10 significant QQIs were detected across the genome with percentages of variance explained (PVE) by the interaction between 0.04 and 10.5% and a total of 20.8% when adding the significant interactions (Table 3, Table S10). Days to maturity had similar behavior with 10 epistatic interactions in different chromosomes and PVE between 1.49 and 6.98% (total 32.5%). Lodging had one significant QQI between Pv02 and Pv10 with a PVE of 2.65% (Table 3). In pigments concentrations, three, six, and one significant QQIs were found for totChl, ChlB, and carotenoids, respectively, with PVE by the interactions ranging from 2.70% in totChl to 6.34% in ChlB (Table 3). Four significant epistatic interactions were found with PVE between 1.6 and 5.96% for yield (total PVE 17.6%) (Table 3, Table S10).

**Table 3:** Top significant epistatic associations identified for agronomic traits

Trait	Pv Chr 1	Marker ID 1	Pv Chr 2	Marker ID 2	Var	R^2^ (%)	-Log10 (*p*)	Valuable allelic combination	Observed mean phenotype	Diff*
Days to flowering (Days)	4	S04_28518734	1	S01_32037216	5.51	10.5	16.29	Alt—Alt	42.33	− 4.51
	3	S03_18855558	4	S04_7449312	4.11	7.8	12.98	Alt—Alt	46.43	− 0.42
	1	S01_30697546	3	S03_17853060	0.19	0.36	12.13	Alt—Alt	42.33	− 4.51
Days to maturity (Days)	2	S02_6535059	3	S03_16330427	1.08	2.2	6.53	Ref—Ref	88.73	− 5.05
	2	S02_18560283	8	S08_62138641	1.61	3.2	6.14	Ref—Alt	81.50	− 12.28
	1	S01_13383398	11	S11_49588787	1.16	2.3	6.09	Alt—Ref	92.72	− 1.06
Lodging (score 1–10)	2	S02_49670245	10	S10_29741404	0.14	2.6	6.45	Alt—Ref	2.66	− 2.23
Total Chlorophyll (µg/mL)	6	S06_25494650	10	S10_22598645	0.33	3.3	6.88	Ref—Alt	37.15	2.39
	2	S02_42197514	3	S03_41954216	0.27	2.7	6.80	Ref—Alt	35.44	0.68
	1	S01_48731617	3	S03_13525186	0.37	3.7	6.37	Ref—Alt	36.25	1.49
Chlorophyll b (µg/mL)	6	S06_25481349	10	S10_10568782	0.33	6.1	6.33	Ref—Alt	15.01	2.11
	1	S01_46894049	2	S02_13541213	0.17	3.2	6.24	Ref—Alt	14.80	1.90
	8	S08_17896781	10	S10_13982000	0.31	5.7	5.04	Alt—Ref	14.23	1.34
Carotenoids (µg/mL)	5	S05_6830869	7	S07_997064	0.03	6.3	5.18	Ref—Alt	5.15	0.62
Yield (Kg/Ha)	3	S03_12432389	8	S08_10888982	3702.0	6.0	7.69	Ref—Ref	1117.64	− 181.5
	4	S04_4394151	4	S01_49862482	3227.6	5.2	6.09	Alt—Ref	1533.21	233.99
	2	S02_47246309	8	S08_33184145	3033.3	4.9	5.50	Alt—Alt	1105.36	− 193.8

Var.: Variance associated with each QQI

Diff.: Difference between the observed mean phenotype for the proposed valuable allelic combination and the overall mean from the combined multi-year BLUPs

**Fig. 4:**
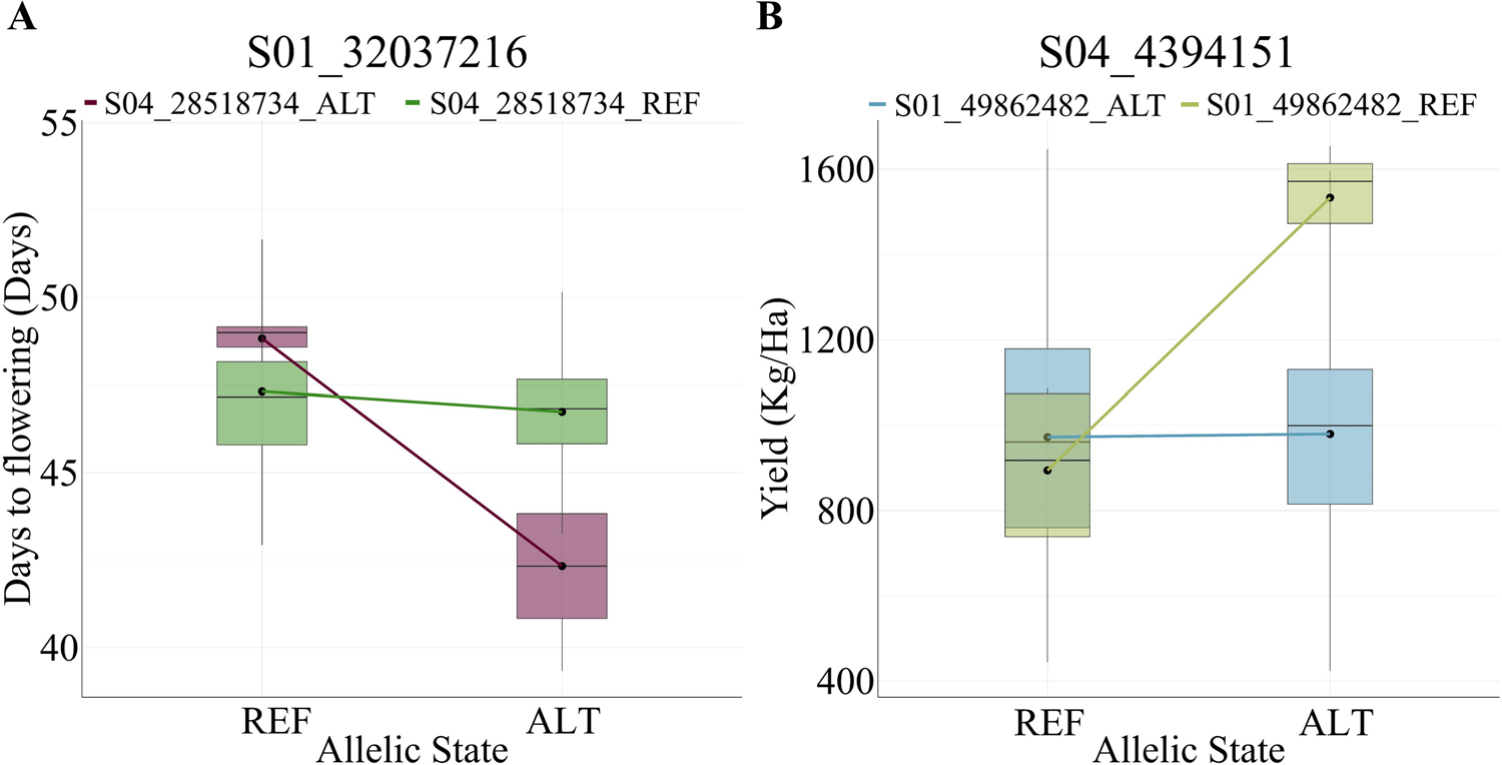
Example of epistasis for flowering time (**A**) and yield (**B**) with the interactions explaining the higher percentage of variance explained. REF denotes the reference allele and ALT is the alternative allele in the variant calling against the common bean reference genome v2.1. Black dots denote the mean and horizontal bars the median of the BLUP-corrected phenotypes for each allelic state combination

The PVE was related to the magnitude (absolute value) of differences observed in the phenotype when the epistatic SNPs changed the allelic state. For instance, in the QQI with the highest PVE in flowering (10.5%), when the SNP S01_32037216 changes from the reference to the alternative allele, no major differences are observed for DTF (around 47 days). However, when the SNP S04_28518734 is considered, the change of allele from reference to alternative in both SNPs causes a reduction of DTF from 48 to 42 DTF (Fig. 4A).

As for yield, a QQI between S01_49862482 on Pv01 and S04_4394151 on Pv04 explained the 5.19% of the phenotypic variance (Table 3). When S04_4394151 is considered alone, the observed yield remains at 975 kg/Ha independent of the allelic state. When both SNPs are present in the alternative allele, the average yield increases to 1,533 kg/Ha (Fig. 4B). Depending on the interaction and the allelic state, the QQI may represent an increase or reduction of yield compared to the overall mean (Table 3). Interestingly, when the markers for the QQI between Pv01 and Pv04 for yield are as reference and alternative, respectively, yield has the potential to increase 234 kg/ha (Fig. 4B, Table 3). Reductions compared to the mean were observed for the other two interactions. The calculated means for each genotypic state (reference or alternative) of the interacting SNPs in the significant QQIs can be found in Table S11. Multiple candidate genes were identified for each of the significant markers in the QQI (Figure S4, Table S10-11). Their potential involvement in the control of the traits is discussed.

## Discussion

### Agronomic traits analysis

In common beans, the key traits valued in the development of new varieties include short flowering and maturity periods, upright architecture, disease resistance, and high yield (Rocha et al. 2019). Similar behaviors and correlations among the evaluated agronomic traits have been previously reported for common bean. Contrasting to our results, Kamfwa et al. (2015), and Moghaddam et al. (2016) reported negative correlations between DTF and yield. However, when studying the relationship among several agronomic traits, Scully and Wallace (1990) found that DTF (varying from 20 to 70 days) and yield are better correlated in a quadratic fashion, with yield increasing until 50 DTF and then drastically decreasing. This may explain the negative, but not considerably high correlation we observed. As expected, DTF and DTM were positively correlated with each other, and lodging had a negative correlation with yield (Resende et al. 2018; MacQueen et al. 2019). Regarding lodging, an upright architecture (type I and II) favors the mechanical harvest, reducing losses and soil-borne diseases compared to prostrate (type II) plants.

Higher concentrations of chlorophylls are well known to be positively correlated to photosynthetic activity (Buttery and Buzzell 1977). ChlB is primarily present in the light-harvesting antenna complex of the photosystem II (PSII) (Croft and Chen 2018), being a potential target trait for plant improvement. ChlA had a very low estimated heritability (5%). In a study of common bean pigments, with the same extraction method, Leitão et al., (2021) also found near zero H^2^ for ChlA. Varying estimations of H^2^ for a trait may depend on the growing conditions, but largely on the phenotyping method used (Pattee et al. 1993). Therefore, the ethanol extraction method or the calculation of the concentrations may not be ideal for ChlA compared to the other pigments. However, further testing, data transformation, and validation may be necessary.

Crop yield is the primary target for plant breeding, but it is also a highly complex trait controlled by multiple genes with minor effects that may change under different environmental conditions. Intriguingly, we observed a weak, but negative correlation between pigment content and yield. No clear trends have been reported in the literature between these two traits. For instance, in a study of 11 genotypes of long bean (*Vigna unguiculata* (L) Walp.), Syukur and Sari Dewi (2019) found positive correlations of pigments with some yield components such as pod size and weight, but a higher pigment content was associated with a lower pod number and seed weight. It is important to mention the potential G × E effect on the observed phenotypes that we tried to control with the experimental design, replications, and the calculation BLUPs. Stability and the G × E dynamics for agronomic and root traits within subsets of the MDP have been previously explored and may contribute to the further selection of materials (Hoyos-Villegas et al. 2016).

### Single-trait Genome-wide association studies

In the evaluated genotypes from MDP, the races Mesoamerica and Durango were observed in the PCA (Figure S2), consistent with previous reports for the panel (Moghaddam et al. 2016; Hoyos-Villegas et al. 2017). In the QQ plots from FarmCPU, the observed *p* values followed the expected distribution with deviations only for the lower *p* values (Figure S3A-G) suggesting that the model properly fitted the phenotypes and identified significant associations in both individual years and the combined analyses. For a few datasets—specifically, the pigment content in certain years and the 3-year yield dataset—slight deviations from the diagonal were observed in the middle *p* value range. However, lower *p* values (higher –Log(*p*)) returned to the expected distribution before reaching significant deviations. Based on these favorable results, the FDR correction for multiple testing was applied. Only SNPs passing the adjusted threshold were further analyzed with higher confidence as candidate SNPs and genes. Multi-locus models such as FarmCPU have demonstrated to be superior compared to single-locus models with a better fitting and lower false discovery rate (Merrick et al. 2022; Sahito et al. 2024). FarmCPU allows the detection of low-frequency variants with significant effects by controlling for large effect loci as fixed effects (Liu et al. 2016; Miao et al. 2018).

The discovery of positive alleles accelerates and enhances the identification of promising germplasm and parents, as well as the selection of progenies (Tibbs Cortes et al. 2021). The estimated PVE and effects (Table 1) show promising contributions of the identified markers for breeding applications such as MAS. After validation in independent populations and diverse backgrounds, sensitive markers based on SNPs such as Kompetitive Allele Specific PCR (KASP) or other fluorescence methods can be developed. These markers allow the rapid screening of potential parents carrying the allele of interest for future cycles, crosses, and introgression efforts. In early generations, molecular markers represent a valuable tool for selection of the offspring for traits with high heritability (Collard and Mackill 2008). More recently, the inclusion of GWAS results and candidate QTNs in prediction models have been explored. For instance, Sehgal et al. (2020) and Chen et al. (2023) demonstrated that the preselection of QTL markers and their inclusion in genomic prediction models increased the prediction ability for grain yield in bread wheat and budburst stage of Norway spruce.

Significant SNPs for flowering time have also been identified on Pv01 and Pv11 in independent experiments on middle American beans (Oladzad et al. 2019b). The candidate model *Phvul.001G158700* encodes a C2H2-type zinc finger domain-containing transcription factor. The homologous gene in Arabidopsis (*AT3G23130*) is the *Floral Defective 10 (FLO10)* or *SUPERMAN (SUP)* gene. SUP is known for being a crucial floral-specific gene that regulates major genes during flower development (homeotic genes) and could be under epigenetic control (Schultz et al. 1991; Bondada et al. 2020). On Pv09, the candidate gene *Phvul.009G039000* is annotated as a F11O4.3-RELATED gene. However, its homologous gene in Arabidopsis (*AT4G35580*) is a NAC transcription factor-like 9. NAC transcription factors are known to be involved in several development stages, placing a potential role of this gene in the control of flowering. For instance, several NAC transcription factors have been reported to be involved in flowering time coordination in soybean (*Glycine max)* (Fraga et al. 2021).

Regarding DTM, Moghaddam et al., (2016) also identified candidate loci on Pv04, but in different positions. The gene *Phvul.001G124700* is annotated as a methyltransferase protein. The homologous in Arabidopsis (*AT2G41380*) encodes a S-adenosyl-L-methionine-dependent methyltransferases superfamily protein that is mainly expressed in senescent leaves (Waese et al. 2017), and it is a potential target of the ATAF1 transcription factor which is involved in the control of senescence and stress responses (Zhang et al. 2023a, b, c). Like in flowering, another member of the NAC (NAM, ATAF1/2, and CUC2) transcription family was identified. The gene, *Phvul.001G023400,* is a No apical meristem (NAM) protein that may have a potential role in the control of maturity in common bean (Podzimska-Sroka et al. 2015; Fraga et al. 2021).

Lodging had strong signals with high PVE that were in line with the highest estimated H^2^ (0.72). The identified loci agreed with previous reports. In middle American beans, Moghaddam et al. (2016) and Hoyos-Villegas et al. (2017) also identified the significant locus on Pv07 as a main controller of plant lodging. Among the candidate genes, *Phvul.007G221500* is a syntaxin of plants SYP7 (SYP7) and the mutation of its homologous gene in Arabidopsis (*AT3G09740*) has been recently shown to have severe effects on plant cell wall structure and components (Zhang et al. 2023c). On Pv06, the gene *Phvul.006G074600* encodes a WRKY transcription factor 33 (WRKY33). WRKY factors have diverse roles in plant development and defense. A paralog of WRKY33, WRKY23, has been reported to be involved in auxin-dependent and independent root development (Grunewald et al. 2012). The role of the genes in cell wall structure and root development may be related to the ability of the plant to keep an upright architecture and prevent lodging.

For chlorophyll content, 12 candidate genes were annotated as present or related to chloroplasts (Table S7). Five genes had homologous genes in Arabidopsis belonging to the cytochrome P450 family. Cytochrome P450 proteins have been shown to participate in the biosynthesis and degradation of chlorophylls (Chakraborty et al. 2023; Yang et al. 2023). Regarding carotenoids, a candidate gene for the SNP S07_31201555, *Phvul.007G192100*, is homologous of the NAC074 protein in Arabidopsis and is proposed to regulate the catabolism of chlorophylls during plant senescence (Xia et al. 2018) which could lead to the accumulation of carotenoids. On Pv11, the gene *Phvul.011G182000* is a WD40-repeat-containing domain protein. In *Medicago truncatula*, a gene of the same family, *MtWD40-1*, regulates the carotenoid biosynthesis genes in flowers when targeted by the MYB activator White Petal 1 (WP1) (Meng et al. 2019), highlighting a potential role of the gene in pigment accumulation in beans. When studying the genetic control of photosynthesis-related traits in beans, Leitão et al. (2021) also identified as the same candidate genes on Pv10 but for net CO_2_ assimilation. Further validation of these genes may elucidate roles in photosynthesis-related traits.

Plant yield is a highly complex trait that depends on multiple genes and their interactions. Several genes related to the control of transcription were identified as candidates. On Pv02, the genes *Phvul.002G121400* and *Phvul.002G121500* are related to the ATP-dependent Switch/Sucrose non-fermentin (SWI/SNF) chromatin remodeling complex. This complex is involved in the regulation of the transcription of genes like those involved in the chromatin memory in response to stress (Meng et al. 2019; Thouly et al. 2020). The transcription factor identified as candidate on Pv05 (*Phvul.005G101900*) encodes a squamosa promoter-binding-like protein 4-related (SPL4). SPL proteins are widely involved in plant growth and development, including the leaf initiation rate and flower and fruit development (Chen et al. 2010; Shalom et al. 2015; Ma et al. 2022) which suggests a likely role of the homologous in common bean in yield-related traits. Another transcription factor associated with the significant SNPs on Pv01 in year 1 was the transcription initiation factor TFIID subunit 10 (TAF10). TAF10 is a ‘selective’ factor that is transiently expressed only in lateral roots, rosettes and floral organs in Arabidopsis. TAF10 targets genes related to meristem and leaf development and *taf10* mutants show phenotypic changes in growth (Tamada et al. 2007). The homologous of TAF10 in common bean may be involved in the overall growth and development of the plant and indirectly influence yield.

### Multi-trait Genome-wide association studies

Multi-trait GWASs have been traditionally conducted by co-localization of significant SNPs from the individual study of phenotypes (Foley et al. 2021). Here, we exploited a MTMM and multi-year data for the joint analysis of pairs of phenotypes to identify QTNs with pleiotropic effects. The MTMM method evaluates variance components within-trait and between-trait simultaneously between correlated phenotypes (Korte et al. 2012). Consistent with the observed correlations (Figure S1), interaction (marginal) or common (significant) effects were detected. In the model evaluation, the QQ plots showed that the observed *p* values followed the expected distribution without deviations from the diagonal (Figure S3H-I). This indicates that no lack of power or model inflation that led to over-estimation and false-positive associations were observed. Only sharp, clear deviations for very high –Log(*p*) values were observed, corresponding to the potential significant associations identified.

For the interaction detected between DTF and yield on Pv02, the gene *Phvul.002G122400* encodes a Decapping 5-like protein (DCP5-L)-related protein. In Arabidopsis, DCP5, a homolog of DCP5-L, has been shown to regulate the transcription of the floral repressor Flowering locus C (FLC) by interacting with the Sister of FCA (SSF) protein (Wang et al. 2023). At the same time, DCP5 is a translational repressor of genes encoding for storage proteins (Xu and Chua 2009). This suggests a potential involvement of DCP5-L in the regulation of both flowering time and yield in common bean. However, further validations are required. A locus on Pv03 with interaction effects between DTF and yield was previously reported in common bean, but through the identification of co-localized significant SNPs (Oladzad et al. 2019a).

The loci detected on Pv04 with common effects for DTM and lodging had candidate genes belonging mainly to the UDP-Glycosyltransferase (UGT) superfamily protein (e.g., *Phvul.004G035800*) and the transducin/WD40 repeat-like superfamily protein (e.g., *Phvul.004G035400)* that have potential roles in the control of both traits (Table S9). The Arabidopsis gene *AT5G15550* is homologous for the bean genes belonging to the WD40 repeat-like superfamily. This gene is implicated in root development through its interaction with the AtPES protein (Zografidis et al. 2014), which may indicate a role in the upright growth habit. Despite not having information about the specific genes belonging to the UGT superfamily, other UGTs have been reported to be upregulated during the senescent stage of Arabidopsis and cotton plants (Rehman et al. 2018).

### Genome-wide epistasis analysis

Over the last decade, GWASs have significantly contributed to the study of the genetic architecture of traits in model and crop plants. However, one of the constraints of conventional GWAS is the limited inclusion and detection of loci with epistatic effects that may explain a significant percentage of the observed variance in different traits (Niel et al. 2015). A few efforts have been developed to model and test interactions with varying levels of success (Sun et al. 2014). The 3VmrMLM approach we adopted is based on the control of polygenetic backgrounds (the influence of several small-effect QTNs) and analysis of variance (ANOVA) for the identification and estimation of QQIs effects (Li et al. 2022). Since no individual SNPs are analyzed in the model (multi-locus), QQ plots are not possible for model evaluation. The confidence in the results for significant and suggested interactions are based on three main parameters: (1) The control for confounding effects such as kinship, population structure, and polygenic backgrounds; (2) A stringent Bonferroni correction to declare significance; and (3) a three-step process for the estimation of variance components and selection of significant QQIs. In the three-step process, the model scans the genome to select for potentially associated QTNs. Then, the identified QTNs are jointly analyzed in a multi-locus model and their effects are estimated using empirical Bayes. Here markers with non-zero effects are further evaluated using a likelihood ratio test (LRT). Finally, significant QQIs should only be declared in a biological context where candidate genes can be identified for each marker. In the method development and validation, Li et al (2022) demonstrated how 3VmrMLM can identify previously reported gene-by-gene interactions in the vicinity of significant QQIs in rice. It is important to highlight that as in any other GWAS study, empirical validation of candidate genes and their interactions must be conducted. Here we intended to develop a first genome-wide scanning and provide candidate markers and genes for epistatic effects in common bean that can be further investigated.

The evaluation of epistatic interactions for the studied traits allowed the identification of pairs of SNPs and candidate genes. Interestingly, at least 14 candidate genes in different pairs of SNPs are annotated in the common bean genome as transcription factors (Table S10), which is highly expected when epistatic interactions are detected (Zheng et al. 2010).

The identified interactions provide novel knowledge and may allow improving simulations, selections, and predictions with models that consider previous information on relevant loci as discussed above (Kaler et al. 2022; Lin et al. 2023). For instance, in yield, the interaction between S01_49862482 and S04_4394151 provides as candidate genes a gibberellin-regulated protein 12-related (*Phvul.001G247600*) and the Transcription factor TCP13 (*Phvul.004G037700*), respectively. TCP13 has been demonstrated to repress gibberellin-regulated proteins such as GA20ox2 in response to shade (Son et al. 2023). In potato (*Solanum tuberosum* L.), Bao et al. (2019) observed that several TCP transcription factors are also regulators in the gibberellin signaling pathway and plant defense. Moreover, the mutation of *stTCP23* resulted in morphological changes in tubers and plant height. Thus, the identified candidate genes may interact to promote plant growth and resistance and contribute to plant performance and yield in common bean.

Due to computational limitations, only a subset of 1,978 markers, resulting in 1,955,253 interactions, was analyzed in a QTN-QTN interaction framework. We acknowledge that filtering and dataset reduction may have excluded important loci. Advancements in computational tools that enable the analysis of more markers within reasonable processing times are still needed and will help uncover new epistatic interactions. In such cases, larger datasets with less stringent filters (e.g., *r*^2^ > 0.3) could be considered. Currently, computationally efficient and reliable methods for higher-order interactions are lacking. As sequencing and bioinformatics advance, approaches capable of modeling third-order interactions (QTN-QTN-QTN) will provide deeper insights into gene interplay, particularly for complex traits like agronomic characteristics.

## Conclusions

A robust phenotypic evaluation of middle American beans paired with multiple approaches allowed a deeper understanding of agronomic traits in common bean. To our knowledge, this is the first study in common bean that simultaneously applies tools beyond single-trait GWAS to identify genomic loci controlling complex trait variation. The multiple analyses performed with data from multiple years allowed the identification of previously reported, but also new candidate markers and genes. Remarkably, new loci were identified to control variation in both flowering and yield, and maturity and lodging in a pleiotropic action. When further validated in external populations or though bulk sequencing methods, and considering the environmental effects, the identified SNPs and candidate genes provide a valuable resource for the selection and crop improvement. For instance, in yield, markers with positive effects up to 91 kg/Has in the combined analyses and 216 kg/Ha in the single-year study may represent a great contribution to the slow increase in yield. In the pleiotropy analyses, the SNPs identified with interaction and common effect on Pv02 and Pv4 would allow the simultaneous improvement of traits. Selecting for the reference allele in S02_26051524 has the potential to maintain and even increase yield by 164 kg/Ha while reducing the flowering time. Similarly, moving toward the ideal upright plant with short maturity periods, keeping the reference allele for the locus on Pv04 or incorporate it in germplasm where it is absent, will be advantageous. Potential epistatic interactions among genomic regions contributing to the variation of traits were also comprehensively identified. The identified epistatic interactions offer the opportunity to refine the stacking of positive alleles. For instance, if considering the interacting loci on Pv03 and Pv08 for yield, keeping both as reference would be beneficial. On the contrary, keeping the markers on Pv01 and Pv04 as reference and alternative, respectively, would show an increase in yield. Overall, our results expand the available fundamental and applied knowledge for common bean improvement that could be utilized in breeding methods such as market-assisted selection, modeling and genomic prediction.

## Supplementary Information

Below is the link to the electronic supplementary material.

Supplementary file1 (XLSX 135 KB)

Supplementary file2 (JPG 836 KB)

Supplementary file3 (JPG 910 KB)

Supplementary file4 (JPG 501 KB)

Supplementary file5 (JPG 461 KB)

Supplementary file6 (JPG 1123 KB)

## Data Availability

Data used for the analyses and scripts are available at: https://github.com/McGillHaricots/peas-andlove/tree/master/AgronomicTraitsGWAS_Epistasis

## Abbreviations

3mrMLM: Three-variance Multi-locus Random-SNP-effect Mixed Linear Model
ANOVA: Analysis of Variance
BLINK: Bayesian information and linkage disequilibrium iteratively nested keyway
BLUP: Best Linear Unbiased Predictor
ChlA: Chlorophyll A
ChlB: Chlorophyll B
DTF: Days to Flowering
DTM: Days to Maturity
FarmCPU: Fixed and random model Circulating Probability Unification
FDR: False Discovery Rate
GBS: Genotype-by-Sequencing
GP: Genomic Prediction
GWAS: Genome-wide Association Analysis
GxE: Genotype-by-Environment
H^2^: Broas-sense heritability
K: Kinship
KASP: Kompetitive Allele Specific PCR
LD: Linkage disequilibrium
MAS: Marker-Assisted Selection
MDP: Mesoamerican Diversity Panel
MLM: Mixed Linear Models
Mt-GWA: Multi-trait GWAs
MTMM: Multi-trait Mixed Model
PCA: Principal Component Analysis
PVE: Phenotypic Variance Explained
Q: Population Structure
QQI: QTN-by-QTN Interactions
QTL: Quantitative Trait Loci
QTN: Quantitative Trait Nucleotide
RBD: Randomized Block Design
St-GWA: Single-Trait GWAS
TotChl: Total Chlorophyll
