# Supplementary figures and images for: Genetic architecture of ideotype-related traits in middle American beans through single trait, multi-trait and epistatic genome-wide analyses
Source: Theor Appl Genet. 2025 Jun 1;138(6):131. doi: 10.1007/s00122-025-04924-w (PMC12127234; doi:10.1007/s00122-025-04924-w)

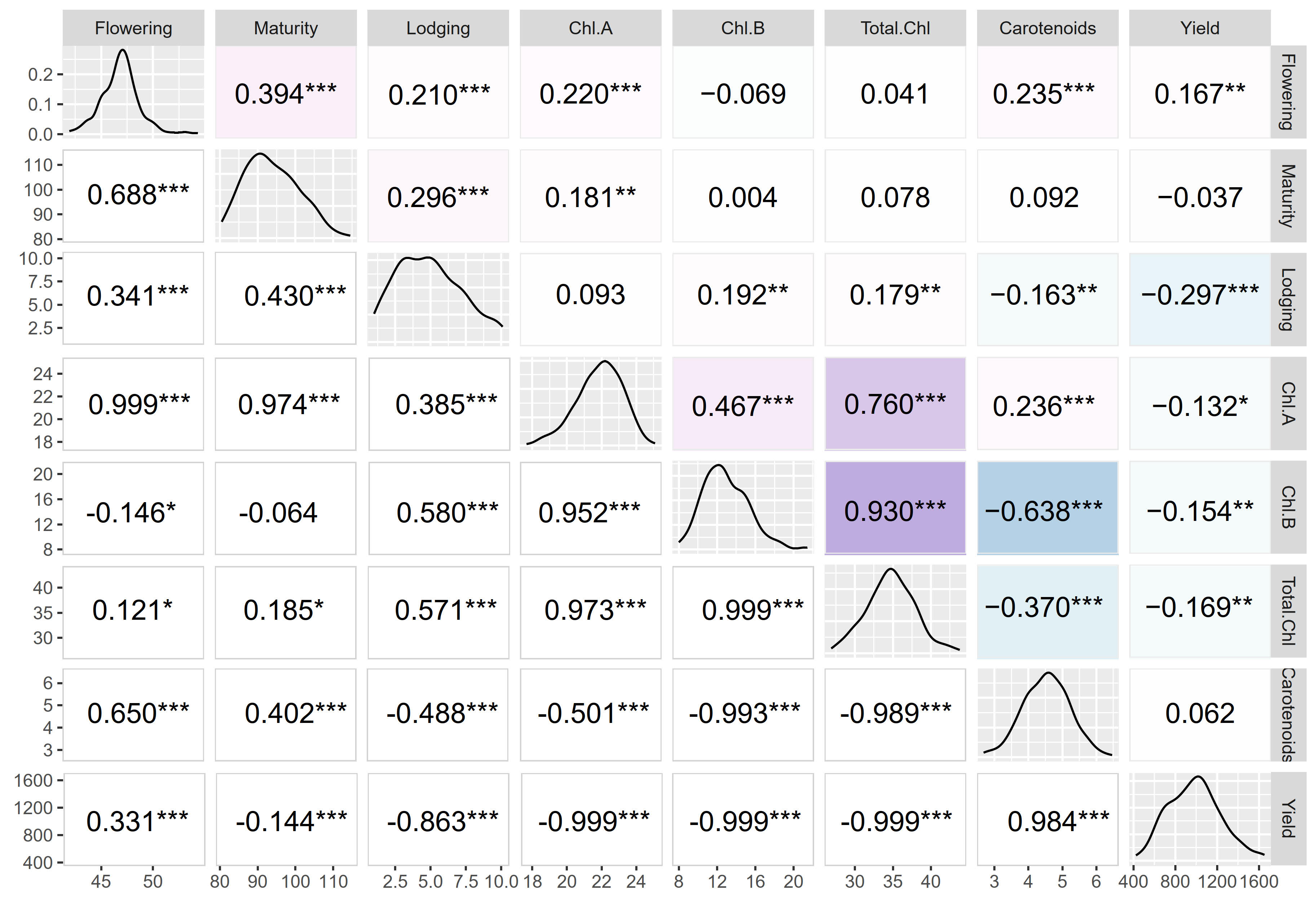

Supplement: Supplementary file 2 — Supplementary file2 (JPG 836 KB) [file 122_2025_4924_MOESM2_ESM.jpg]

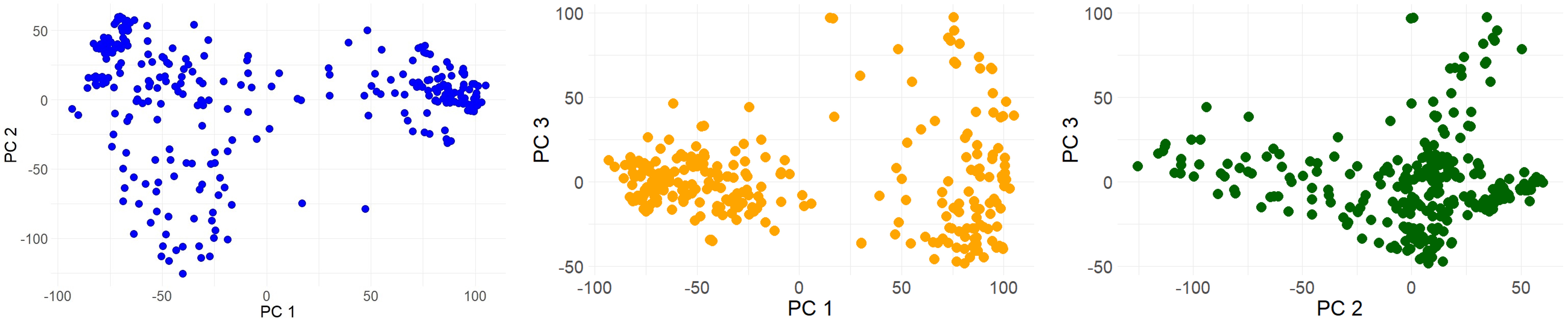

Supplement: Supplementary file 3 — Supplementary file3 (JPG 910 KB) [file 122_2025_4924_MOESM3_ESM.jpg]

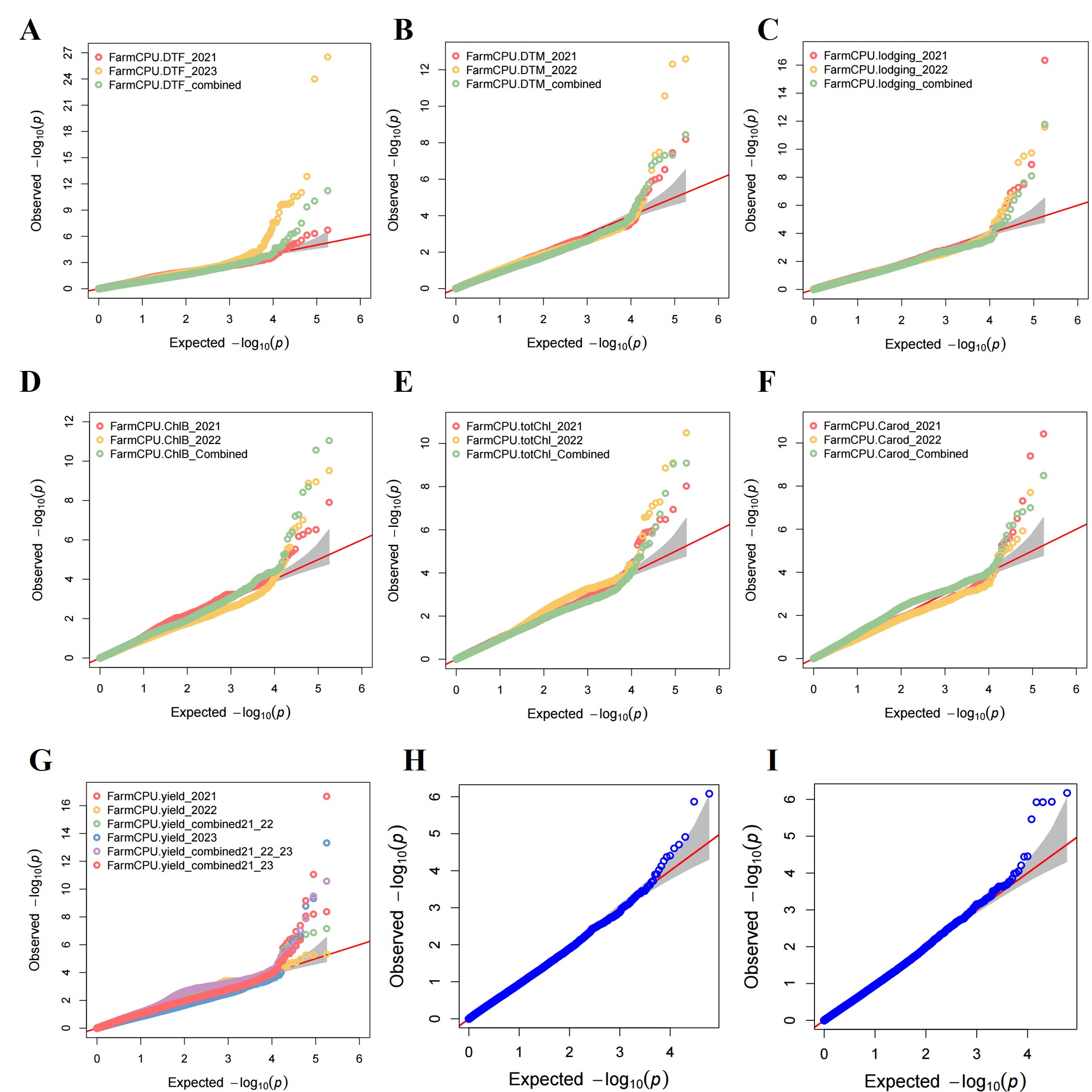

Supplement: Supplementary file 4 — Supplementary file4 (JPG 501 KB) [file 122_2025_4924_MOESM4_ESM.jpg]

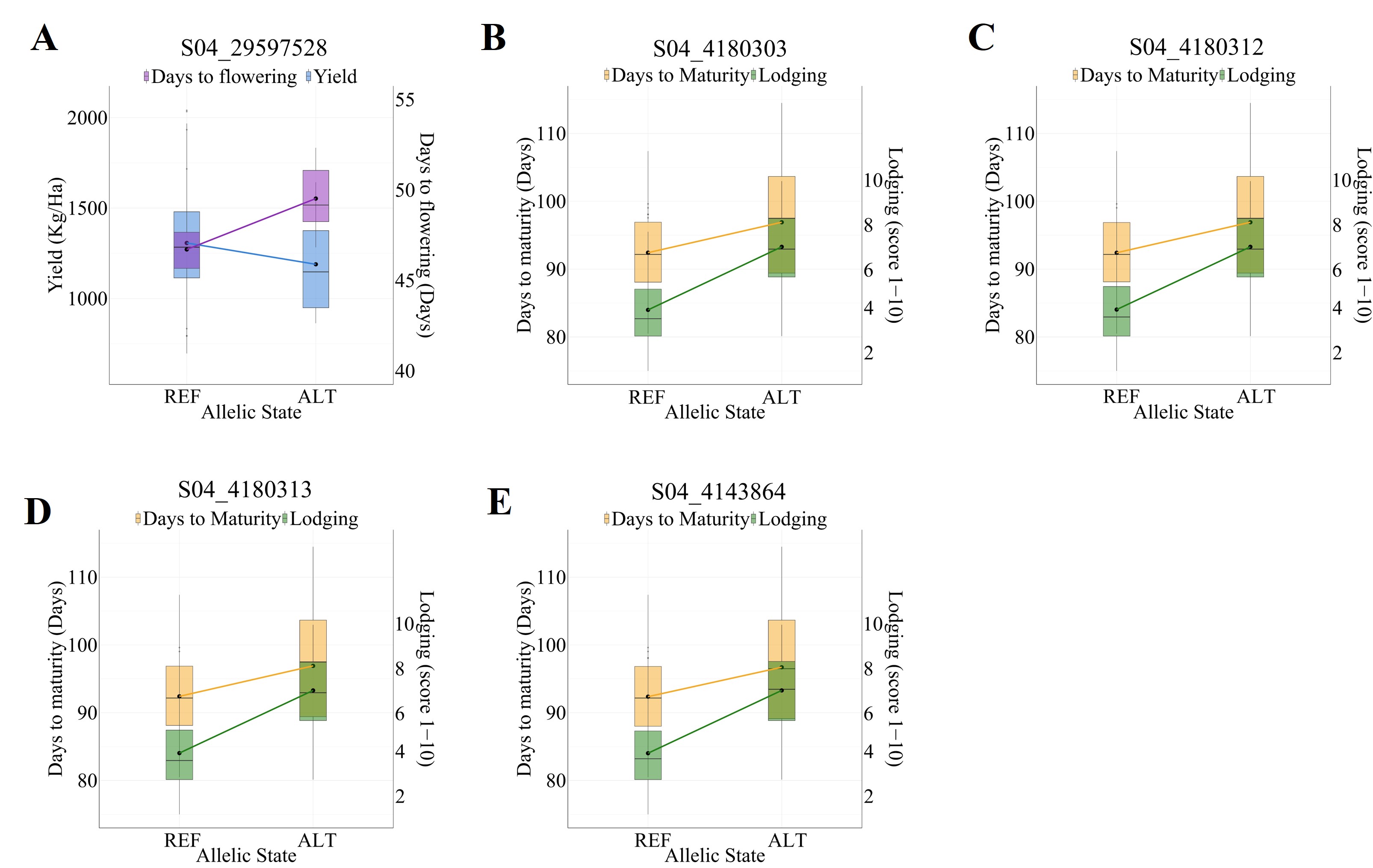

Supplement: Supplementary file 5 — Supplementary file5 (JPG 461 KB) [file 122_2025_4924_MOESM5_ESM.jpg]

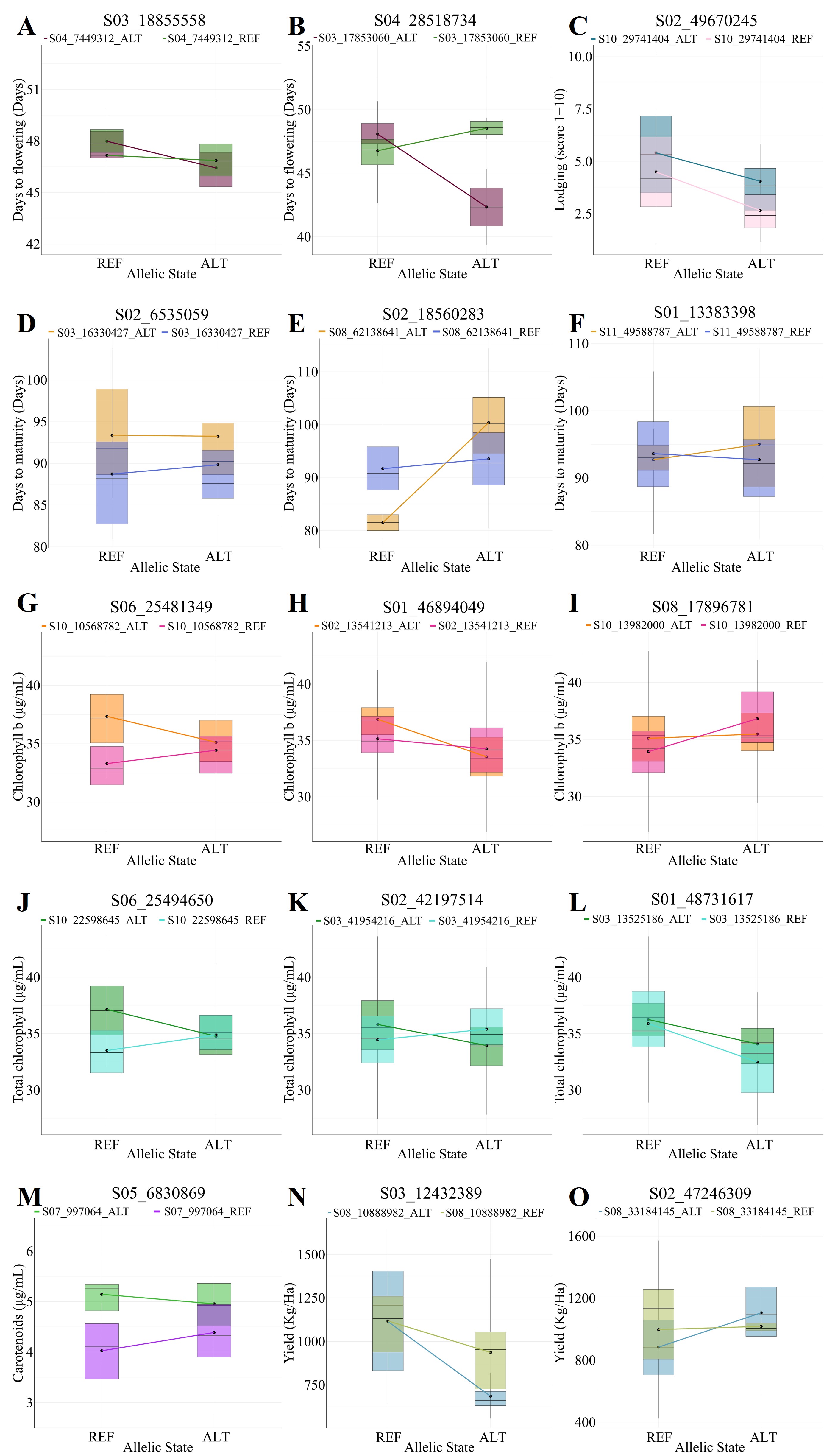

Supplement: Supplementary file 6 — Supplementary file6 (JPG 1123 KB) [file 122_2025_4924_MOESM6_ESM.jpg]
